# Five-year Contraceptive Use of 52-mg Levonorgestrel Releasing Intrauterine System in Young Women, Menstrual Patterns, and New Contraceptive Choice

**DOI:** 10.1055/s-0043-1776032

**Published:** 2023-11-29

**Authors:** Elaine Cristina Fontes de Oliveira, Ana Luiza Lunardi Rocha

**Affiliations:** 1 Departament of Obstetrics and Gynecology, Universidade Federal de Minas Gerais, Belo Horizonte, MG, Brazil

**Keywords:** levonorgestrel intrauterine system, adolescents, amenorrhea, menstruation, contraception, long-acting reversible contraception, sistema intrauterino de levonorgestrel, adolescentes, amenorreia, menstruação, contracepção, contracepção reversível de longa duração

## Abstract

**Objective** To evaluate the continuation rates of the 52-mg levonorgestrel-releasing intrauterine system (LNG-IUS) during the first 5 years of use, reasons for its discontinuation, bleeding patterns, and new contraceptive choice after the 5 ^th^ year, in adolescents and young women.

**Methods** The present study was a 5-year prospective cohort conducted in a Family Planning Service of a tertiary hospital in Brazil. We selected 100 healthy women between 15 and 24 years old who used 52-mg LNG-IUS for contraception. The clinical follow-up of these women took place from June 2017 to December 2022. The study evaluated the continuation rates of the method, reasons for its discontinuation, bleeding patterns, and new contraceptive choice after the 5 ^th^ year. Continuous data were reported as mean ± standard deviation (SD) and range (minimum-maximum). Categorical variables were described as percentages.

**Results** The continuation rates of LNG-IUS were 89.1% (82/92), 82.9% (72/87), 75.3% (64/85), 70.5% (60/85), and 64.2% (54/84) in the 1 ^st^ , 2 ^nd^ , 3 ^rd^ , 4 ^th^ , and 5 ^th^ years of use, respectively. The main reason for discontinuation was acne (11/30). Amenorrhea rates were 50, 54.1, 39, 35.7, and 51.8% at 12, 24, 36, 48, and 60 months, respectively. All patients who completed the study and needed contraception after the 5 ^th^ year opted for long-acting contraceptive methods (LARC).

**Conclusion** The LNG-IUS showed high continuation rates in adolescents and young women in the first 5 years of use. Most patients who completed the study chose a LARC method after the 5 ^th^ year.

## Resumo

**Objetivo** Avaliar as taxas de continuação do sistema intrauterino liberador de levonorgestrel (SIU-LNG) 52 mg durante os primeiros 5 anos de uso, razões de sua descontinuação, padrões de sangramento e nova escolha contraceptiva após o 5° ano, em adolescentes e mulheres jovens.

**Métodos** O estudo foi uma coorte prospectiva de 5 anos realizada em um Serviço de Planejamento Familiar de um hospital terciário no Brasil. Selecionamos 100 mulheres saudáveis entre 15 e 24 anos que usaram o SIU-LNG 52 mg para contracepção. O acompanhamento clínico dessas mulheres ocorreu de junho de 2017 a dezembro de 2022. O estudo avaliou as taxas de continuação do método, razões de sua descontinuação, padrões de sangramento e nova escolha contraceptiva após o 5° ano. Os dados contínuos foram relatados como média ± DP e intervalo (mínimo-máximo). As variáveis categóricas foram descritas como porcentagens.

**Resultados** As taxas de continuação do SIU-LNG foram 89,1% (82/92), 82,9% (72/87), 75,3% (64/85), 70,5% (60/85) e 64,2% (54/84) no 1°, 2°, 3°, 4° e 5° anos de uso, respectivamente. O principal motivo de descontinuação foi a acne (11/30). As taxas de amenorreia foram de 50, 54,1, 39, 35,7 e 51,8% aos 12, 24, 36, 48 e 60 meses, respectivamente. Todas as pacientes que completaram o estudo e necessitaram de contracepção após o 5° ano optaram por métodos contraceptivos de longa duração (LARC).

**Conclusão** O SIU-LNG apresentou altas taxas de continuação em adolescentes e mulheres jovens nos primeiros 5 anos de uso. A maioria das pacientes que completou o estudo escolheu um método LARC após o 5° ano.

## Introduction

Teenage pregnancy is a severe global health problem, especially in low- and middle-income countries. 1 In most countries, the median age at first intercourse is around 17 years old. By 15 years of age, ∼ 15% of females had had intercourse; by 18 years, ∼ 60%, and by 20 years of age, ∼ 80%. 2 In adolescents, contraceptive use and sexual activity can affect the pregnancy rate. 3 Thus, the prescription of contraceptive methods that are acceptable and effective is specially necessary for this group.

The use of long-acting contraceptive methods (LARC) in adolescents could lead to the prevention of pregnancies at an early age and the spacing between them, avoiding unintended pregnancies and abortions. 4 Intrauterine devices (IUDs) are safe for these women, with meager rates of complications such as pelvic inflammatory disease (PID) and uterine perforation. 5 6 Despite the safety of IUDs in adolescents and nulliparous women and recommendations of many different medical societies for their use, they are still underused by women < 20 years old. 7 8 9 Between 2017 and 2019, 38.7% of women aged 15 to 19 years old in the United States were currently using contraception. The most common contraceptive method used by these women is contraceptive pills, and it was estimated that only 5.8% of young women had used LARC. 10

Several studies have demonstrated high continuity rates for using the 52-mg levonorgestrel-releasing system (LNG-IUS) among young women. 11 12 13 Counseling candidates about possible bleeding patterns and adverse effects could lead to lower IUD discontinuation rates. 11 Contraceptive counseling should include anticipatory guidance for adolescents and their parents regarding possible menstrual changes (such as lighter bleeding, spotting, or amenorrhea), side effects (such as acne, headaches, nausea, breast tenderness, and mood changes), and noncontraceptive benefits such as management of irregular or abnormal uterine bleeding and treatment of dysmenorrhea. 14

Regarding the side effects of LNG-IUS, some studies demonstrated that participants aged between 16 and 35 years old at enrollment were significantly more likely to report new or worsening acne, dyspareunia, pelvic pain, and dysmenorrhea. 15

Barriers to using LARC by adolescents include patients' lack of familiarity with or understanding of the methods, potentially high cost of initiation, lack of access, low parental acceptance, and obstetrician-gynecologists' and other health care providers' misconceptions about the safety of LARC use in adolescents. 16

The present study aimed to evaluate continuation rates, bleeding patterns, reasons for its continuation, and new contraceptive choices in the 5 ^th^ year of use of the 52-mg LNG-IUS in adolescents and young women.

## Methods

The present study was a 5-year prospective cohort conducted at the Family Planning Service, Department of Obstetrics and Gynecology, Hospital das Clínicas of the Universidade Federal de Minas Gerais (UFMG, in the Portuguese acronym), Belo Horizonte, state of Minas Gerais, Brazil, from June 2017 to December 2022. The project was approved by the Research Ethics Committee of UFMG (protocol 65138816.8.0000.5149). Participants were women who sought the Family Planning service for 52-mg LNG-IUS insertion for contraception or treatment of gynecological conditions. All women who agreed voluntarily to participate in the study signed an Informed Consent Form (ICF). Participants < 18 years old provided informed assent, while their parents provided the ICF. The present study is the continuation of a previously published paper with a 5-year follow-up. 17

The study was conducted with nulliparous or parous women aged between 15 and 24 years old who were eligible for the use of LNG-IUS, according to the World Health Organization (WHO) medical eligibility criteria for contraceptive use. The exclusion criteria were: uterine sounding < 5 cm; cervical cytological abnormalities in the last 18 months; uterine cavity distortion (any congenital or acquired uterine abnormality distorting the uterine cavity in a manner that is incompatible with IUD insertion); current breast cancer, endometrial cancer, or cervical cancer (awaiting treatment); recent history of pelvic inflammatory disease or untreated genitourinary tract infection; abnormal uterine bleeding of unknown cause; < 6 weeks postpartum or postabortion.

Women were applying for the use of LNG-IUS for planning counseling. They accepted the LNG-IUS as a contraceptive method after an explanation of all contraceptive methods available. They answered a questionnaire containing information on education level, parity, previous menstrual pattern, and previous use of contraception. A gynecologist performed a clinical examination. It was not necessary to perform a gynecological ultrasound prior to the insertion of the devices.

The insertion of the 52-mg LNG-IUS was performed up to the 7 ^th^ day of the menstrual cycle by an obstetric gynecologist and/or a trained resident physician. A urinary or blood pregnancy test was used to exclude pregnancy if the woman was not using an effective contraceptive method. For patients using any effective method of contraception, the LNG-IUS was inserted at any time of the menstrual cycle. According to the service's routine protocol, a transvaginal ultrasound was performed to verify the LNG-IUS positioning 30 days after insertion. The LNG-IUS was considered malpositioned when described as partially expelled, rotated, embedded in the myometrium, or located in the lower uterine segment or cervix.

Follow-up visits occurred every year up to 5 years of use. The continuation rates of the method, reasons for its discontinuation, and bleeding patterns were evaluated. The menstrual pattern was self-reported and classified according to the following definitions. Menstrual cycles within 24-32 days, with bleeding lasting no more than 5 days, were considered regular cycles. Amenorrhea is the absence of uterine bleeding for at least 3 months. Spotting was considered occasional and unpredictable bleeding in small amounts. When it was impossible to return to face-to-face consultations, the women were contacted and questioned via telephone or social networks. At the end of the 5 ^th^ year of follow-up, patients were contacted by telephone to choose a new contraceptive method since, in Brazil, extended use of the 52-mg LNG-IUS has not yet been approved by the National Health Surveillance Agency (ANVISA).

Continuous data were reported as mean ± standard deviation (SD) and range (minimum-maximum). Categorical variables were described as percentages.

## Results

We performed 52-mg LNG-IUS insertion in 100 adolescents and young women. The characteristics of the participants are presented in Table 1 . This group's mean (± SD) age was 22 ± 1.9 years old (range 16 to 24 years old). Most participants were nulliparous (86%). The main reason for the placement of LNG-IUS was contraception (96%). Other reasons for LNG-IUS placement were: treatment of dysmenorrhea (1%), desire for amenorrhea (1%), history of thrombosis (1%), and others (1%). The main contraceptive method previously used by these women was combined hormonal contraceptives (60%). Other contraceptive methods used by these patients were progestin-only pills (4%), combined hormonal injectables (6%), lactational amenorrhea (2%), vaginal rings (1%), condoms (18%), or none (9%).

**Table 1:** Demographic and gynecological variables ( *n* = 100)

Variables	Total
Age (years old)	22.2 ± 1.9
Minimum–Maximum	16.0–24.0
Race	
White	65(65.0)
Not white	35(35.0)
Weight (kg)	60.5 ± 11.3
Height (cm)	1.62 ± 0.1
BMI (kg/m ^2^ )	
Low weight	4 (4.0)
Normal	75 (75.0)
Overweight	14 (14.0)
Obese	5 (5.0)
Parity	
Nulliparous	86 (86.0)
Previous cesarean	1 (1.0)
Previous normal delivery	12(12.0)
Any prior abortion	1 (1.0)
Education	
Less than high school degree	8 (8.0)
High school degree	14 (14.0)
Some college or higher	78 (78.0)
Uterine sounding	7.26 ± 0.74

Abbreviations: BMI, body mass index.

Data are presented as *n* (%) or mean ± SD.

**Fig. 1:**
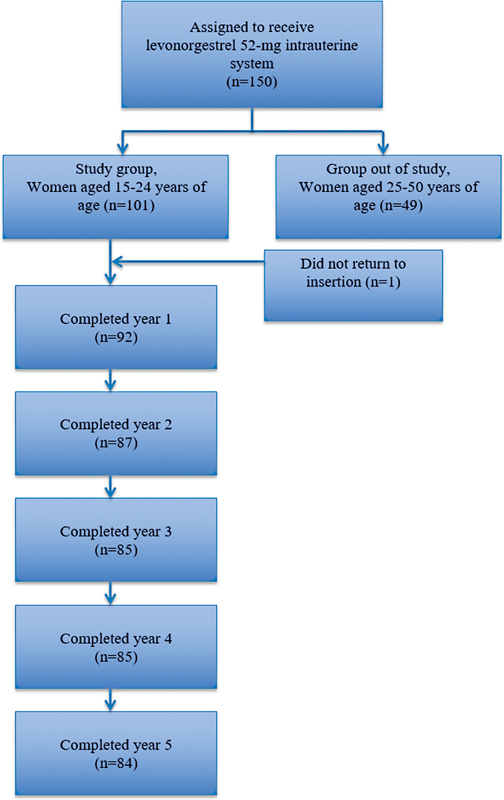
Participants flowchart.

The only complication observed during the insertion of LNG-IUS was vasa-vagal responses (such as dizziness, nausea, and vomiting) in 7% of women. Major complications, such as uterine perforation or infection, did not occur. Five insertions were guided by ultrasound. Ninety-two LNG-IUS were well positioned, 6 were poorly positioned, and 2 patients did not return for reassessment after insertion. Patients with poorly positioned IUS had their IUS repositioned, or a new device was inserted. Participants' follow-up over the first 5 years of the present trial is presented in the Participants Flowchart ( Fig. 1 ).

As shown in Table 2 , among those who were not lost to follow-up, the 52-mg LNG-IUS continuation rates were 89.1% (82/92), 82.8% (72/87), 75.3% (64/85), 70.5% (60/85), and 64.2% (54/84) in the 1 ^st^ , 2 ^nd^ , 3 ^rd^ , 4 ^th^ , and 5 ^th^ years of use, respectively.

**Table 2:** Continuation rates of 52-mg levonorgestrel-releasing intrauterine system in 5-year follow-up of young women ( *n* = 100)

Time (years)	Numbers of removals at the end of the year	Losses	Continuation rates (%)
1	10	8	89.1 (82/92)
2	15	13	82.8 (72/87)
3	21	15	75.3 (64/85)
4	24	15	70.5 (60/85)
5	30	16	64.2 (54/84)

**Fig. 2:**
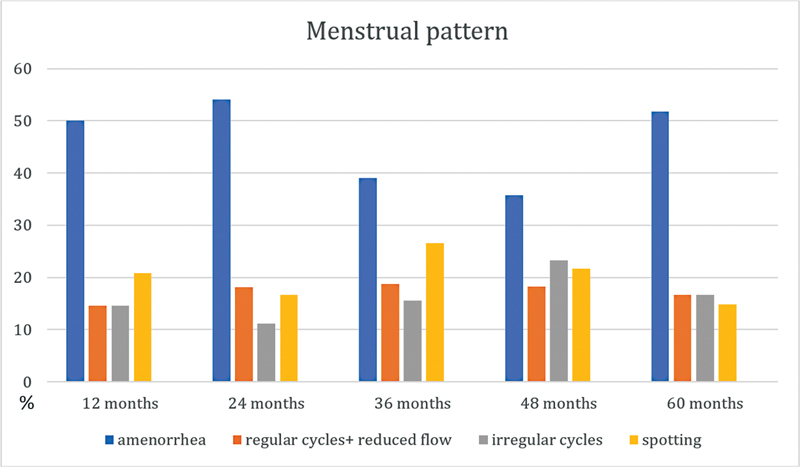
Menstrual patterns in females using a 52-mg levonorgestrel-releasing intrauterine system at 5-year follow-up.

Menstrual patterns through the 5 years of observation are presented in Figure 2 . Amenorrhea rates were 50, 54.1, 39, 35.7, and 51.8% at 12, 24, 36, 48, and 60 months after insertion. Spotting rates varied during the 5 years of use (20.8, 16.7, 26.6, 21.7, and 14.8%, respectively). Some patients who reported spotting in the 4 ^th^ year had amenorrhea in the 5 ^th^ year, and patients with amenorrhea were less likely to discontinue SIU-LNG use in the last year.

Percentage of different bleeding patterns presented by adolescents and young women using 52-mg LNG-IUS during 12, 24, 36, 48, and 60 months of follow-up. In total, 30 participants stopped using LNG-IUS either because of adverse events or a desire to become pregnant. The reasons for the discontinuation of 52-mg LNG-IUS and the time at which they occurred are presented in Table 3 . The main reason for discontinuation was acne (11/30).

**Table 3:** Reasons for discontinuation of the 52-mg levonorgestrel-releasing intrauterine system among young adults in 5 years of follow-up

Time (months)	Reason
1	expulsion
1	bicornuate utero
1	poor positioning of IUS
2	expulsion
4	dyspareunia
5	recurrent candidiasis
5	poor positioning of IUS
6	ruptured ovarian cyst
6	acne
11	acne, loss of hair
15	acne
17	acne
19	dysmenorrhea
21	expulsion
22	irregular uterine bleeding
24	depressive symptoms
27	acne
27	abdominal pain
32	depressive symptoms
32	acne, dysmenorrhea
35	get free of hormones
40	loss of hair; dyspareunia
40	acne
45	previous venous trombosys
46	acne
49	desire to conceive
50	acne
54	acne, loss of hair
54	acne
58	anxiety

Abbreviation: IUS, intrauterine system.

At the end of the 5 ^th^ year, 23 patients chose extended use of 52-mg LNG-IUS, 24 chose to insert a new 52-mg LNG-IUS, three chose to change to 19-mg LNG-IUS, and 2 chose to insert a copper intrauterine device. Two patients chose not to use any contraceptive methods because they were without a male partner ( Table 4 ).

**Table 4:** Methods selected by young women after the 5 ^th^ year of using the 52-mg levonorgestrel-releasing intrauterine system ( *n* = 54)

Method selected	n
Insertion of new 52-mg levonorgestrel-releasing intrauterine system	24
Extended-use of 52-mg levonorgestrel-releasing intrauterine system	23
19-mg levonorgestrel-releasing intrauterine system	3
Copper intrauterine device	2
None	2

## Discussion

The present prospective cohort study describes continuation rates, menstrual patterns, and reasons for discontinuation of 52-mg LNG-IUS among Brazilian adolescents and young adults and new contraceptive choices after the 5 ^th^ year.

There was no device insertion failure, and in case of difficulty, the devices were inserted guided by ultrasound. The authors believe it is important to emphasize that it is unnecessary to perform an ultrasound prior to device insertion since the incidence of uterine malformations in the population is low. In our study, no patient had a previous ultrasound, and only one patient had a uterine malformation identified on the ultrasound performed after insertion. Similar to several previous studies, there were no serious complications such as ectopic pregnancy, pelvic inflammatory disease, or uterine perforation. 5 6

Our study found high LNG-IUS continuation rates among young women. Among those women who were not lost to follow-up, the 52-mg LNG-IUS continuation rates were 89.1% (82/92), 82.8% (72/87), 75.3% (64/85), 70.5% (60/85), and 64.2% (54/84) in the 1 ^st^ , 2 ^nd^ , 3 ^rd^ , 4 ^th^ , and 5 ^th^ years of use, respectively. Continuation rates for IUDs are generally higher compared with other contraceptive methods for women aged ≤ 25 years old. A previous systematic review showed that the 12-month continuation rate of IUD users was 86.5% in adolescents and young women. 18 A retrospective cohort that evaluated continuation rates of the 52-mg LNG-IUS in the general population found continuation rates for the entire group of 85, 77, 70, 64, and 58 per 100 women years at 1, 2, 3, 4, and 5 years after insertion, respectively. 11

The main reason for the discontinuation of LNG-IUS in this group was acne, which contrasts with previous studies. 6 11 15 Younger patients are more likely to report acne onset or worsening, possibly due to the higher incidences of acne in this age group. 15 19 In addition, our study's patients who had previously used oral contraceptives were more likely to report worsening acne (RR 1.68). Furthermore, progestin contraceptive therapies have also been associated with acne development. As suggested by previous studies, the levonorgestrel intrauterine device may exacerbate inflammatory acne. 20 21 However, there was no questioning or classification of the patient's acne before the insertion of the method.

The discontinuation rate due to irregular bleeding was extremely low (only 1 patient), and most patients had favorable bleeding patterns during the 5-year follow-up. Amenorrhea was the predominant bleeding pattern throughout the 5 years. Amenorrhea rates were 50, 54.1, 39, 35.7, and 51.8% at 12, 24, 36, 48, and 60 months after insertion. The increase in the amenorrhea rate is justified by the fact that some patients who reported spotting in the 4 ^th^ year had amenorrhea in the 5 ^th^ year, and patients who had amenorrhea were less likely to discontinue SIU-LNG use in the last year. This menstrual pattern coincides with descriptions in the literature. 22

The expulsion rate in our study was 2.2% at 12 months and 3.6% after an overage follow-up of 60 months. This is consistent with other studies supporting that younger women are less likely to experience expulsion than older women. 6 23

It is important to observe that all women who needed contraception after the 5 ^th^ year opted to maintain a LARC method. Because of the high risk of unintentional pregnancy, adolescents need highly effective contraceptive methods.

A criticism of the methodology used in the present study is the subjective evaluation of blood loss, so we cannot assure that the menstrual pattern was correctly described. Another problem is that we did not question acne presence before SIU-LNG insertion.

Teenagers and young women are more likely to request premature discontinuation of their IUDs for contraception. 6 Researchers believe that having a trained team capable of answering frequently asked questions from patients, as well as treating side effects, could lead to a reduction in device discontinuation rates. In our study, patients were instructed to contact the main researcher at any time.

The strengths of our study include data collection by a single investigator throughout the five-year follow-up period, as well as lower loss (16%) to follow-up during this period. All patients had a contact telephone number for the main researcher during the entire follow-up period of the research, which may have positively interfered with the low loss of follow-up.

The data from the present study contribute to reaffirming the safety of LNG-IUS in young patients, as well as the high continuation rate during 5-year use. Special attention should be given to counseling and treatment of adverse effects, especially acne.

## Conclusion

The LNG-IUS should be routinely offered to young women as a safe and effective contraceptive option. The LNG-IUS showed high continuation rates in adolescents and young women in the first 5 years of use. Most patients had a favorable menstrual pattern with the use of 52-mg LNG-IUS. Young patients should be counseled about side effects, especially acne. Most patients who completed the study chose a LARC method after the 5 ^th^ year.
